# Emblic Leafflower (*Phyllanthus emblica* L.) Fruits Ameliorate Vascular Smooth Muscle Cell Dysfunction in Hyperglycemia: An Underlying Mechanism Involved in Ellagitannin Metabolite Urolithin A

**DOI:** 10.1155/2018/8478943

**Published:** 2018-03-06

**Authors:** Junxuan Zhou, Cong Zhang, Guo-Hua Zheng, Zhenpeng Qiu

**Affiliations:** ^1^College of Pharmacy, Hubei University of Chinese Medicine, Wuhan 430065, China; ^2^Key Laboratory of Chinese Medicine Resource and Compound Prescription, Ministry of Education, Hubei University of Chinese Medicine, Wuhan 430065, China

## Abstract

Ellagitannins in* Phyllanthus emblica* L. (emblic leafflower fruits) have been thought of as the beneficial constituents for ameliorating endocrinal and metabolic diseases including diabetes. However, the effect of emblic leafflower fruits on diabetic vascular complications involved in ellagitannin-derived urolithin metabolites is still rare. In this study, acetylcholine-induced endothelium-independent relaxation in aortas was facilitated upon emblic leafflower fruit consumption in the single dose streptozotocin-induced hyperglycemic rats. Emblic leafflower fruit consumption also suppressed the phosphorylation of Akt (Thr308) in the hyperglycemic aortas. More importantly, urolithin A (UroA) and its derived phase II metabolites were identified as the metabolites upon emblic leafflower fruit consumption by HPLC-ESI-Q-TOF-MS. Moreover, UroA reduced the protein expressions of phosphor-Akt (Thr308) and *β*-catenin in a high glucose-induced A7r5 vascular smooth muscle cell proliferation model. Furthermore, accumulation of *β*-catenin protein and activation of Wnt signaling in LiCl-triggered A7r5 cells were also ameliorated by UroA treatment. In conclusion, our data demonstrate that emblic leafflower fruit consumption facilitates the vascular function in hyperglycemic rats by regulating Akt/*β*-catenin signaling, and the effects are potentially mediated by the ellagitannin metabolite urolithin A.

## 1. Introduction

Diabetes is an endocrine disease diagnosed by pathological blood glucose elevation which is caused by absolute (Type I) or relative (Type II) insulin insufficiency [1]. Type I diabetes which resulted from an irreversible autoimmune damage of pancreatic *β* cells accelerates the disturbance of carbohydrate metabolism, which subsequently causes a series of complications in patients with diabetes mellitus, including nephrosis, neuropathy, or cardiovascular disease. Though the commercial insulin injection is a primary strategy for blood glucose control, the protection for organ functions is still indispensable in the therapeutic process of diabetes. The continuous hyperglycemia plays a crucial role in both the diabetic macroangiopathy and microangiopathy [2]. Moreover, the vascular dysfunction contributes to high mortality in diabetic patients, and hypofunction of the endothelium may be an initial event in this disease [3].

The elevated glucose level induces dysfunction of vascular endothelium, recruits inflammatory cells to the impaired endothelium, and disorganizes the expression of several protooncogenes, which synergistically trigger the vascular remodeling [4]. Also, diabetes is correlated with the superabundant production of reactive oxygen species (ROS) by hyperglycemia, which contributes to the elimination of endothelium-derived relaxing factor nitric oxide (NO) and the accumulation of endogenous vasoconstrictor substances, such as endothelin-1 [5, 6]. Both* in vitro* and* in vivo* evidence demonstrate that high glucose condition obstructs acetylcholine-mediated endothelium-dependent relaxation (EDR) and interdicts the generation of NO [7]. From this respect, the vasomotor function may also be implicated in hyperglycemia vascular remodeling. Therefore, to improve vascular function is beneficial for diabetes treatment.

The pathological proliferation or hypertrophy is not beneficial for vascular smooth muscle cell (VSMC) function, but nocuous for VSMCs at the diastolic level. It is known that activation of PI3K/Akt signaling is vital for various biological processes including cell proliferation, invasion, and motility behaviors. Particularly, high glucose-induced VSMC proliferation and chemokinesis are involved in the activation of PI3K/Akt and NF-*κ*B signaling pathways [8]. Moreover, canonical Wnt/*β*-catenin signaling which is featured by the accumulation of *β*-catenin and the elevated transcription of downstream oncotargets (c-Myc and cyclin D1) can be mediated by PI3K/Akt crosstalk [9]. Furthermore, small molecule compounds antagonizing Akt phosphorylation [10] and *β*-catenin accumulation [11] suppress the VSMC proliferation, which also provides insights into strategies for alleviating vascular dysfunction of hyperglycemia.

Recently, dietary phenolic constituents including ellagitannins (ETs) have been thought of as supplements for ameliorating cardiovascular diseases or targeting several pathologic changes in the preclinical study [12]. In fact, the main metabolites of ellagitannins in systemic circulation are urolithins, which are beneficial for vascular health [13].* Phyllanthus emblica* L., also called emblic leafflower fruit or Indian gooseberry, naturally occurs in the tropical and subtropical area of Asia such as South China, Burma, and India. Emblic leafflower fruits (ELF) are regarded as food and also applied as the traditional geoauthentic crude drug in Asia. Moreover, the previous study illuminated that ETs and flavonoids were considered as main beneficial components for health in ELF [14]. However, the function of ELF involved in vascular protection is still rare. The present study aims to evaluate the effects of ELF on vascular function in streptozotocin-induced hyperglycemia rats. Furthermore, the effects of ellagitannin metabolite urolithin A from* in vitro* VSMC models were also evaluated.

## 2. Materials and Methods

### 2.1. Chemicals

Urolithin A (UroA) was synthesized according to previously described protocols [15]. Streptozocin (STZ), acetylcholine (ACh), phenylephrine (PE), sodium nitroprusside (SNP), glucose, and lithium chloride (LiCl) were all purchased from Sigma-Aldrich (USA). All other chemicals were of analytical grade.

### 2.2. Preparation of Plant Materials

The emblic leafflower fruits were collected from Jinzao Town, Shantou City, Guangdong Province, China (N23°26′14.71′′, E116°20′36.79′′). The sample (A voucher specimen ZP-003) was authenticated by Dr. Ling Gong in Hubei University of Chinese Medicine. The samples (10 kg) were sliced and then squeezed by a juice squeezer (JYZ-V911, Joyoung, China). The fresh liquid (juice, 2800 mL) was filtered, pasteurized, concentrated, and stored at −18°C until use.

### 2.3. Polyphenol Measurement

Before componential analysis, the juice was lyophilized, and total polyphenol content of ELF was measured spectrophotometrically with phosphomolybdic/phosphotungstic acid reagent [16]. The juice (0.1 L) was freeze-dried to get 2 g of dry powder. The content of EA in ELF was also quantitated by chromatographic comparisons (HPLC-DAD) with reference material. Other ETs in the juice were identified as previously described [14]. The chromatographic condition is as follows: ZORBAX SB-C18 column (Agilent Technologies, US, 250 mm × 4.6 mm, 5 *μ*m), 25°C using acetonitrile (A) and water/formic acid (99.9 : 0.1, v : v) (B) as the mobile phases at a flow rate of 1 mL/min. Protocol: 0 min: 0% A, 100% B; 5 min: 0% A, 100% B; 15 min: 5% A, 95% B; 20 min: 10% A, 90% B; 25 min: 15% A, 85% B; 30 min: 25% A, 75% B; 40 min: 25% A, 75% B; 55 min: 60% A, 40% B; 60 min: 0% A, 100% B.

### 2.4. Animals

Adult male Sprague–Dawley rats weighing 200–240 g were maintained at the humidity-controlled temperature of 22 ± 3°C and in an artificial light/dark cycle (12 h : 12 h). All experimental procedures involved in animals were approved by the Ethics Committee in Hubei University of Chinese Medicine with the license number SYXK-2012-0067.

### 2.5. Experimental Design in Rats

The hyperglycemia model was induced by a single dose of STZ (60 mg/kg, i.v.) injection in overnight fasted rats as previously described [17]. The development of hyperglycemia in rats was identified by the plasma glucose assessment of blood samples collected from the tail vein 72 h after STZ exposure. STZ-injected animals with blood glucose levels > 15.0 mM were considered diabetic. Blood glucose values were collected every week. Four days after STZ administration, rats were randomly grouped as follows: (1) normal control rats (NCR); (2) hyperglycemic rats (HGR); (3) hyperglycemic rats treated with low dose of ELF (LD, ELF 25 g/kg); (4) hyperglycemic rats treated with median dose (MD, ELF 50 g/kg); (5) hyperglycemic rats treated with high dose (HD, ELF 75 g/kg). The ELF consumption in rats was through the juice intragastric administration once a day for five weeks. The doses (ELF 25 g/kg, 50 g/kg, and 75 g/kg) were converted to the weights of original fruit samples (2.8 mL of juice volume/10 g of fruit weight).

During the experimental period, animals were treated with ELF in the general cage and then placed individually in metabolism cages for the collection of urine once a week. After five weeks, rats were weighed, and glucose values in rats were measured. Then diabetic rats were anesthetized with 0.3% pentobarbital sodium (30 mg/kg) and sacrificed by decapitation.

### 2.6. Serum Nitric Oxide (NO) Measurement

The serum was obtained from the whole blood by centrifugation (12000 ×g, 5 min, 4°C). The supernatant was used for total nitric oxide (NO) assessment. Total nitric oxide production was analyzed by using a Nitrate/Nitrite Assay Kit (Beyotime, China).

### 2.7. Isometric Tension Vasomotor Assessment

The aorta was dissected out and transferred to the Petri dish containing Krebs-Henseleit (Krebs') solution. The perivascular tissue was removed from the isolated aorta. Then the aorta ring approximately 4 mm in length was divided from each thoracic aorta and maintained using organ bath chambers containing 10 mL of Krebs' solution in an artificial atmosphere of carbogen (95% O_2_ and 5% CO_2_). The above conditions were also applied to hitch the aortic ring between two stainless steel hooks joint to a tonotransducer.

For studying the vasomotor function, aortic rings were subjected to an optimized load of 1.0 g and the tension was recorded using a data acquisition system (MedLab, Nanjing Medease Co. Ltd., China). The ring response curves to phenylephrine with the cumulative concentration (1 nM–30 *μ*M) were obtained. After prevasoconstriction with phenylephrine (1 *μ*M), the rings were stimulated by acetylcholine (0.001–10 *μ*M) or SNP (0.001–10 *μ*M) with incremental dosing for evaluating the vasodilator response as previously described [18].

### 2.8. Ellagitannin Metabolite Analysis

HPLC-ESI-Q-TOF-MS was applied to identify UroA in urine samples (obtained from diabetic rats treated with high dose ELF) on a ZORBAX SB-C18 column (Agilent Technologies, US, 250 mm × 4.6 mm, 5 *μ*m) at 30°C using acetonitrile (A) and water/formic acid (99.9 : 0.1, v : v) (B) as the mobile phases at a flow rate of 0.4 mL/min. The gradient began with 10% A in B to reach 60% A in B at 35 min and 90% A in B at 38 min. The mass detector was an ion trap mass spectrometer (micrOTOF-Q II, Bruker Daltonics Inc., USA) equipped with an electrospray ionization (ESI) system (capillary voltage 3500 V, dry temperature 180°C). UroA was also identified by its retention time and UV spectrum (full-wavelength scan), and the stratographic occurrence was validated by comparison using the UroA reference.

### 2.9. Cell Culture, Treatment, and Cell Proliferation Assay

A7r5 vascular smooth muscle cells were purchased from American Type Culture Collection (Manassas, VA, USA). Cells were cultured in previously described condition [19]. A7r5 cells were seeded in different plates (96-, 12-, and 6-well) and maintained until 75% confluency. Before UroA treatment, the cells were preincubated with glucose (30 mM) or LiCl (20 mM) for 12 h and 3 h to conduct the HG-induced vascular smooth muscle cell proliferation model or Wnt signaling activated model, respectively. Then, UroA was subsequently added and further incubated for 48 h.

For cell proliferation assay, 10 *μ*L of CCK solution was added to each well, and the mixture was incubated for 1 h at 37°C. The absorbance at 450 nm was measured using a Multiskan FC plate reader and analyzed with Skanlt for Multiscan FC software (Thermo Scientific, USA).

### 2.10. Real-Time Quantification of Messenger RNA (mRNA)

The total cellular RNA was extracted using the TRIzol® reagent (Invitrogen, USA). Oligo (dT) 18 primer was applied to bind poly-A tails in RNA for generating complementary DNA (cDNA). The quantificational polymerase chain reaction (qPCR) was performed on a Mini-Opticon™ (Bio-Rad, USA) using FastStart Universal SYBR Green Master (Roche, USA). After predenaturation at 95°C for 2 min, the transcriptional targets were amplified with 40 cycles at 95°C for 5 s and 55°C for 25 s. The housekeeping gene *β*-actin was employed as an internal control for normalizing the mRNA expression. Primers for qPCR are listed in the Supplemental Materials (Table S2).

### 2.11. Western Blot Assay

Immunoblotting was performed as previously described [20]. After gradient separated by SDS-PAGE and blotted to polyvinylidene fluoride membranes, the expressing levels of target proteins in arteria or A7r5 cells were assessed using a chemiluminescence system with an enhanced chemiluminescence (ECL) reagent (Thermo Scientific, USA). The following primary antibodies were used: phosphor-Akt (Thr308) (CST, Beverly, MA, USA) and *β*-catenin (CST, Beverly, MA, USA). The HRP-conjugated GAPDH antibody (Proteintech, Wuhan, China) served as an internal standard for normalization. The densitometric analysis was performed using Image Lab 4.1 (Bio-Rad, USA).

### 2.12. Luciferase Assay for TOPflash/FOPflash Reporter Activity

For studying the effects of UroA on Wnt signaling, A7r5 cells were seeded in 24-well plates at 5 × 10^4^ cells per well and maintained to approximately 75% confluency. Then cells were cotransfected with TOPflash/FOPFlash (Upstate Biotechnology) and Renilla reporter plasmid pRL-CMV (10 ng) as previously described [21]. After LiCl preincubation and UroA administration, cell lysates were collected, and Renilla luciferase activities were estimated with a dual-luciferase reporter assay system (Promega, USA).

### 2.13. Statistical Analysis

The maximum diastolic values (*E*max) induced by acetylcholine or SNP in the isolated rings were recorded as a percentage of the tension signal in response to phenylephrine (1 *μ*M) treatment. The data were analyzed using SPSS (version 11.5). Values were recorded as mean ± standard deviation (SD). Differences between two and multiple groups were analyzed with Student's *t*-test and ANOVA, respectively. Results with a *P* value < 0.05 (*∗*, #) or 0.01 (*∗∗*, ##) were indicated as statistically significant.

## 3. Results

### 3.1. Polyphenol Content and HPLC-DAD Analysis for ETs and EA in ELF

In the present study, the polyphenol content of three samples collected at the same location within one week was evaluated by the Folin−Ciocalteu assay. The total polyphenol contents were found to vary from 18.12 ± 2.6, 17.82 ± 3.2, to 18.71 ± 1.4% GAE in the freeze-dried powder. No significant differences were found among three batches of fruits.

EA and ETs were identified by the characteristic UV-vis spectra with absorption maxima at 254 and 360 nm in HPLC-DAD analysis (Supplemental Material, Figure S1). Peak C was identified as EA, and the content (0.18–0.25%) in fresh samples was quantified with the standard curve of EA authentic standard. Other peaks considered as ETs (Peak A, putranjivain A; Peak B, chebulagic acid) were provisionally distinguished by comparing with the ultraviolet absorption spectrum data in the previous study [14]. The confirmatory data were present in Supplemental Materials.

### 3.2. Emblic Leafflower Fruit Consumption Improves Endothelium-Dependent Vasodilatation of Aortic Rings from Hyperglycemia Rats

The body weight and postprandial blood glucose values were stated in Supplemental Materials (Table S1). After five weeks, rats in the HGR group had lost weight, compared with the NCR group (^#^*P* < 0.05). From the third week, the postprandial blood sugar values in the HD group were altered with moderate reduction, compared to that in hyperglycemia controls (^*∗*^*P* < 0.05). Regrettably, the difference vanished at the fifth week (Table S1). These data indicated that the effects of ELF on the blood glucose could not be attractive in the present study.

**Figure 1:**
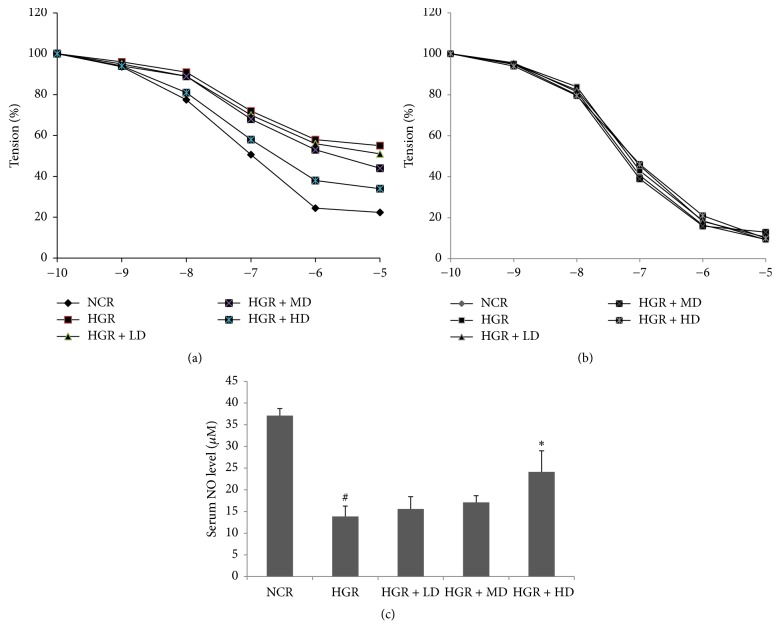
*Emblic leafflower fruit (ELF) consumption ameliorates endothelium-dependent vasodilatation in hyperglycemic rats*. (a) Acetylcholine-induced endothelium-dependent relaxation (EDR), (b) SNP-induced endothelium-independent relaxation (EIR) in rat aortic rings, and (c) serum nitric oxide (NO) levels in (1) normal control rats (NCR); (2) hyperglycemic rats (HGR); (3) hyperglycemic rats treated with low dose of ELF (LD, ELF 25 g/kg); (4) hyperglycemic rats treated with median dose (MD, ELF 50 g/kg); (5) hyperglycemic rats treated with high dose (HD, ELF 75 g/kg). Mean ± SD, *n* = 6. ^#^*P* < 0.05 versus NCR; ^*∗*^*P* < 0.05 versus HGR.

Nevertheless, the endothelium-dependent vasodilatation of aortic rings from hyperglycemia rats was improved by ELF consumption. In Figure 1(a), compared with the HGR group, the level of EDR aroused by ACh in the isolated rings with PE-induced contraction was elevated in the HD group. Compared to the NCR group, *E*_max_ values of the rings decreased to 42.7%  ± 3.5% in the HGR group. The MD and HD consumption of ELF for five weeks ameliorates the vasoreactivity over that in the HGR group and the *E*_max_ values of aortic rings attaining 55.1%  ± 4.6% and 77.5%  ± 2.7%, respectively (*P* < 0.01). Moreover, there was no significant difference among the five groups in the assessment of endothelium-independent relaxation mediated by SNP (Figure 1(b)).

### 3.3. NO Synthesis Is Enhanced upon ELF Consumption in Hyperglycemic Rats

In Figure 1(c), serum NO content was significantly less in the STZ-induced hyperglycemic rats than in the NCR group from the Nitrate/Nitrite Assay (^#^*P* < 0.05). Compared with the HGR group, ELF consumption in the HD group elevated the serum NO level (^*∗*^*P* < 0.05). It should be noted that no difference of the serum NO level was observed between the normal rats with ELF consumption and normal control rats (data not shown). These results indicated that the ELF consumption could alleviate the obstacle of NO synthesis in hyperglycemic rats but not elevate the NO level.

### 3.4. Phosphorylation of Akt and Transcription of Protooncogenes in Hyperglycemia Aortas Are Diminished upon ELF Consumption

**Figure 2:**
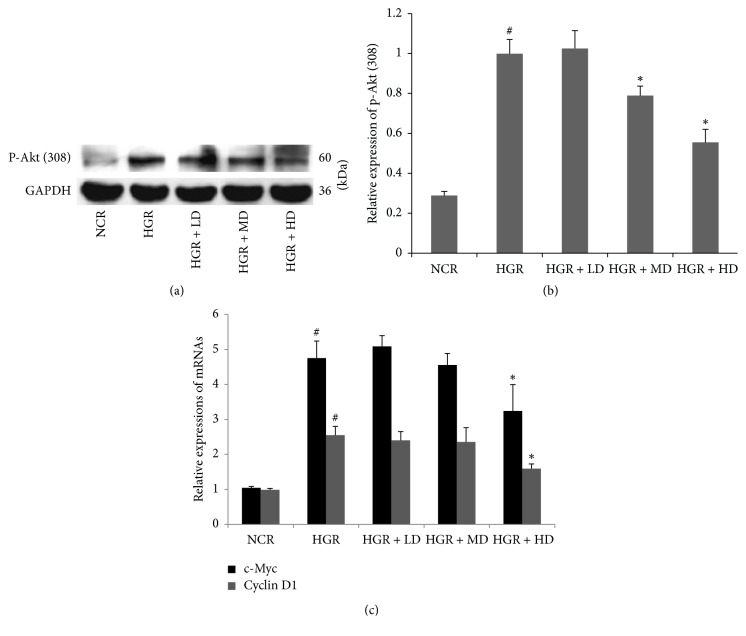
*Emblic leafflower fruit (ELF) administration suppresses the protein expression of phosphorylated-Akt (Thr308) and the mRNA transcriptions of c-Myc and cyclin D1*. The protein expression of phosphorylated-Akt (Thr308) (a, b) and the mRNA transcriptions of c-Myc and cyclin D1 (c) in rat aortic rings in (1) normal control rats (NCR); (2) hyperglycemic rats (HGR); (3) hyperglycemic rats treated with low dose of ELF (LD, ELF 25 g/kg); (4) hyperglycemic rats treated with median dose (MD, ELF 50 g/kg); (5) hyperglycemic rats treated with high dose (HD, ELF 75 g/kg). *n* = 3. ^#^*P* < 0.05 versus NCR; ^*∗*^*P* < 0.05 versus HGR.

In Figures 2(a) and 2(b), the protein expression of Thr-308 phosphorylated-Akt was suppressed in the arteria in the HD group, compared with that of the HGR group (^*∗*^*P* < 0.05). The previous evidence indicated that *β*-catenin accumulation induced by Akt phosphorylation could trigger Wnt/*β*-catenin signaling by binding T cell factor/lymphoid enhancer factor transcription factors. Cyclin D1 and c-Myc, which were considered as protooncogenes mediated by Wnt/*β*-catenin signaling, were downregulated at the mRNA level (qPCR) in the HD group (Figure 2(c)), compared to the HGR group (^*∗*^*P* < 0.05). Therefore, the data suggested that Akt phosphorylation and Wnt/*β*-catenin signaling downstream transcriptional targets could be suppressed by ELF treatment in hyperglycemia aortas.

### 3.5. Ellagitannin Metabolite UroA and Its Derivatives Occurred in Urine upon ELF Consumption

**Figure 3:**
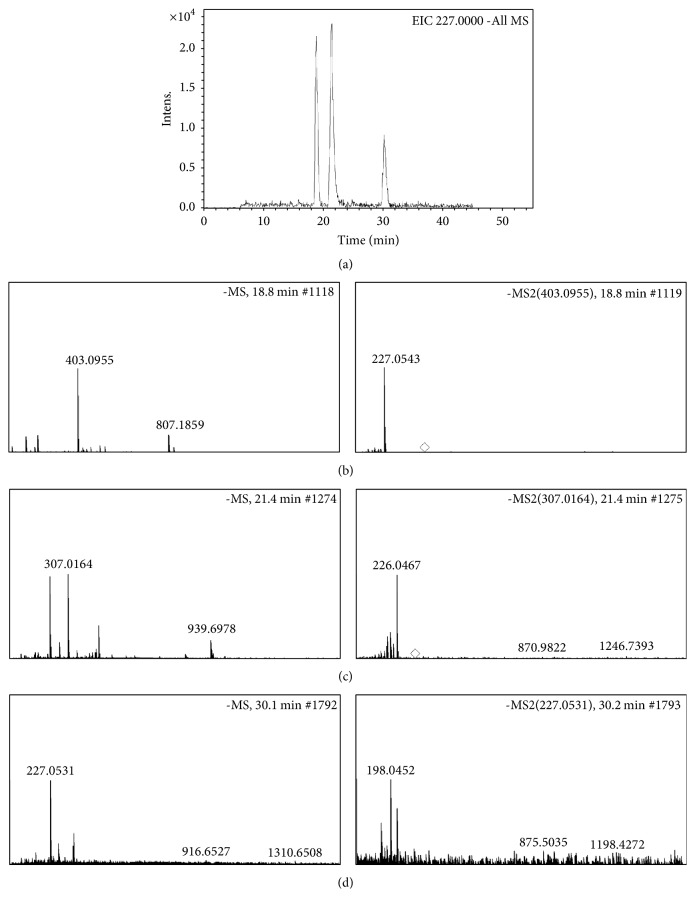
*Urolithin A (UroA) related metabolites were converted from ellagitannins in emblic leafflower fruits in vivo*. The urine samples in hyperglycemic rats treated with high dose of ELF were collected before sacrifice and further analyzed by HPLC-ESI-Q-TOF-MS. The peaks involved in UroA (*m*/*z* 227.0539[M-H]−) in extracted ion chromatogram (EIC) (a) were identified as UroA glucuronic acid (b), UroA-sulfate (b), and UroA (d), respectively.

Because ETs were identified in ELF, it was imperative to confirm whether ETs in ELF could be converted to urolithin derivatives in rats. As shown in Figure 3, three peaks involved in UroA (*m*/*z* 227.0539[M-H]−) in extracted ion chromatogram (Figure 3(a)) were identified as UroA glucuronic acid (Figure 3(b), 18.8 min), UroA-sulfate (Figure 3(d), 21.4 min), and UroA (Figure 3(d), 30.2 min) by HPLC-ESI-Q-TOF-MS assay, respectively. Our data appear to be in agreement with these opinions that urolithin metabolites occur upon consumption of ETs-rich diets including walnuts and pomegranate juice [22].

### 3.6. UroA Suppresses Akt/*β*-Catenin Signaling in a High Glucose-Induced VSMC Proliferating Model

**Figure 4:**
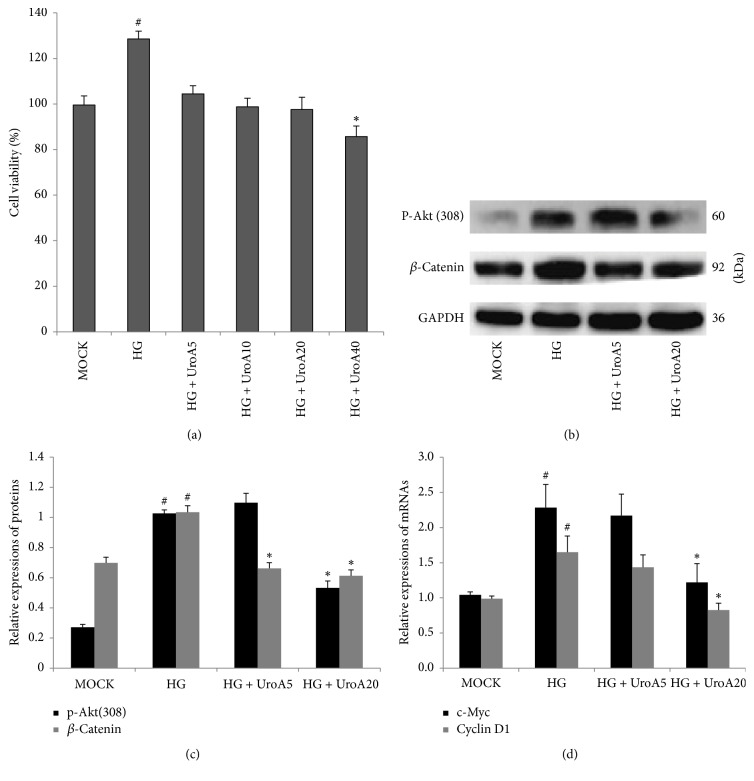
*UroA attenuates high glucose- (HG-) induced A7r5 cell proliferation by suppressing Akt/β-catenin signaling*. (a) The effect of UroA on high glucose- (HG-) induced A7r5 cell proliferation. Cells were seeded in 96 well and preincubated with HG (30 mM) for 12 h. The cells were treated by UroA (5–40 *μ*M). CCK-8 assay was performed to determine cell viability as described in Materials and Methods. (b) Western blotting analysis for the protein expressions of phosphorylated-Akt (Thr308) (60 kD) and *β*-catenin (92 kD) in HG-incubated A7r5 cells. (C) Histograms show phosphorylated-Akt (Thr308) and *β*-catenin protein levels quantified by western blot optical analysis. (c) The effect of UroA on the mRNA expressions of c-Myc and cyclin D1 was quantified by qPCR in HG-induced VSMC proliferating model. Quantitative data are mean ± SD, *n* = 3. ^#^*P* < 0.05 versus MOCK; ^*∗*^*P* < 0.05 versus HG group.

In consideration of vasoactive functions (Figure 1) and UroA metabolites of ELF (Figure 3), the effects of UroA on Akt/*β*-catenin signaling were further evaluated in a high glucose- (HG-) induced A7r5 VSMC model. In Figure 4(a), HG addition elevated A7r5 cell proliferation approximately 0.6-fold at 48 h (^#^*P* < 0.05), which was weakened by UroA treatment (5–40 *μ*M). Because 40 *μ*M UroA treatment led to obvious cell cytotoxicity (^#^*P* < 0.05, compared to the MOCK group) in A7r5 cells, the UroA concentrations from 5–20 *μ*M were used in the following investigation. To further evaluate whether HG-induced cell proliferation was corresponding to the* in vivo* molecular evidence, the expressions of mRNAs and proteins were assessed by qPCR and western blot, respectively. As shown in Figures 4(b) and 4(c), the expressions of Thr-308 phosphorylated-Akt and total *β*-catenin were elevated in the HG-induced group (^#^*P* < 0.05), which was significantly suppressed by UroA (^*∗*^*P* < 0.05). Moreover, the mRNA expressions of the Wnt downstream proliferative targets, c-Myc and cyclin D1, increased in the HG group (^#^*P* < 0.05), which were also reduced by UroA treatment (^*∗*^*P* < 0.05; Figure 4(d)). These results indicated that the inhibiting effects on the HG-induced A7r5 cell proliferation could be involved in suppression of Akt and Wnt/*β*-catenin signaling.

### 3.7. UroA Ameliorates LiCl-Induced Activation of Wnt/*β*-Catenin Signaling in VSMCs

**Figure 5:**
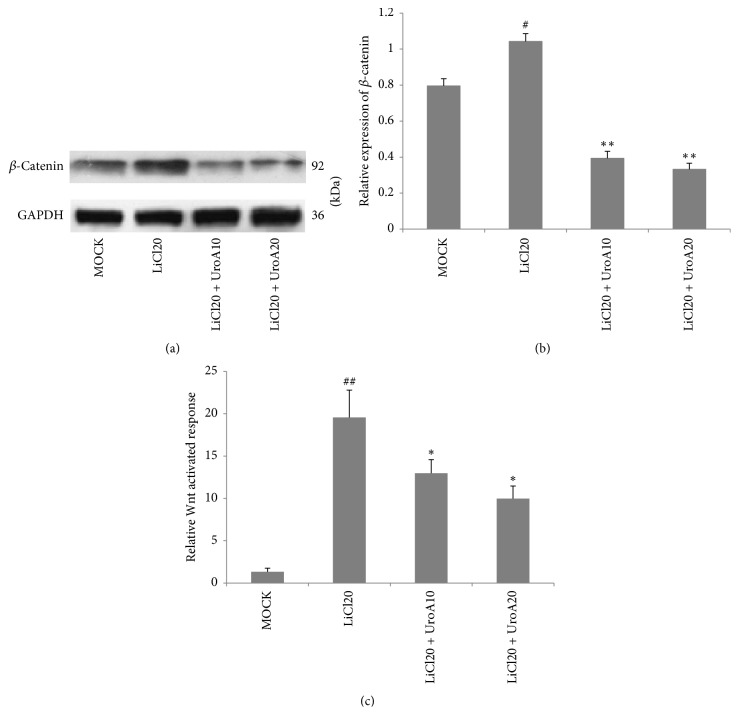
*UroA suppresses LiCl-induced activation of Wnt/β-catenin signaling in A7r5 cells*. (a, b) Cells were preincubated with LiCl (20 mM) for 3 h. Then the cells were further treated with UroA for 48 h. Western blotting analysis was applied for quantifying the protein expression of *β*-catenin (92 kD) in LiCl-pretreated A7r5 cells. (c) UroA suppressed Tcf/Lef-mediated transcriptional activity in A7r5 cells. Cells were transiently cotransfected with TopFlash or FopFlash and Renilla reporter plasmid with Lipofectamine 3000. After posttransfection, cells were treated as described in Materials and Methods. Luciferase values were measured using a dual-luciferase reporter assay system. Quantitative data are mean ± SD, *n* = 3. ^#^*P* < 0.05, ^##^*P* < 0.01 versus MOCK; ^*∗*^*P* < 0.05 and ^*∗∗*^*P* < 0.01 versus LiCl20 group.

Generally, pathological GSK-3*β* dysfunction obstructs the degradation of *β*-catenin by ubiquitination following activation of Wnt signaling [23]. Here, LiCl, the validated GSK-3 inhibitor activating Wnt signaling, was employed to clarify the effects of UroA on Wnt/*β*-catenin signaling in VSMCs. To do so, the conventional medium was altered to media supplemented with LiCl (20 mM) or NaCl (20 mM) (MOCK group) before UroA administration. LiCl treatment elevated the expression of *β*-catenin, compared to the MOCK group (^#^*P* < 0.01). The results showed that LiCl-induced *β*-catenin accumulation was suppressed to 0.4- and 0.3-fold by UroA treatment with 10 and 20 *μ*M, respectively, compared to the LiCl-treated group (^*∗∗*^*P* < 0.01; Figures 5(a) and 5(b)). Moreover, in the TOPflash/FOPflash reporter activity assay, UroA (10 and 20 *μ*M) suppressed the firefly luciferase expression to 0.6- and 0.5-fold, compared to the LiCl-treated group (^*∗*^*P* < 0.05; Figure 5(c)). These data further confirmed that the abnormal activation of Wnt/*β*-catenin signaling in VSMCs could be reduced by UroA.

## 4. Discussion

To prevent and treat noncommunicable chronic diseases has cumulatively become a global concern. Recently, except conventional medical treatments for cardiovascular diseases, developing natural supplements from diet-sources to ameliorate cardiocerebrovascular complications is also urgently demanded. These could be beneficial for improving the survival quality of patients as well as opportunities for the food industry.

*Phyllanthus emblica* L. (ELF), which produced in tropical and subtropical areas including India, Malaysia, and southern China, are the subsidiary agricultural products and commonly consumed as a kind of seasonal fruits in these regions [24]. Moreover, the ELF has been applied as a kind of ethnomedicinal drugs in Sri Lanka, India, and Tibetan autonomous region of China. Functional trials of* in vitro* and* in vivo* suggest that ELF has antioxidant, antidiabetic, antimicrobial, lipid-regulatory, antiproliferative properties, and especially ameliorative effects on cardiovascular disorders [25]. Noteworthy, these pharmacological findings have suggested that the polyphenol compounds, including ETs, in the fruits might be the healthful constituents. However, due to the low bioavailability of ETs and metabolism from ellagitannins to urolithins by intestinal floras, the above evidence obtained from* in vitro* cell model with ellagitannins is still limited.

Recently, phenolic components were identified from ELF by column chromatography combined with nuclear magnetic resonance spectrum. It is acknowledged that gallotannins and ETs are the major phenolic constituents of ELF [26]. Moreover, phenolic constituents in the methanol extract of the fruits were also analyzed by HPLC-DAD and HPLC-ESI(-)-MS [14]. Here, the juice of ELF was lyophilized, and the constituents in juice were assessed according to the available protocols with minor modification (see Materials and Methods). The data indicated that ETs and EA were detectable in the freeze-dried powder. In fact, EA was considered as a catabolite of ETs in the plants (such as acid and enzymatic hydrolysis). The amount of EA in samples also reflects the presence of ETs. Our componential data involved in ETs in this study are accordant to that of other ELF extracts in the other published work [25]. These data preliminarily indicate that ETs could be useful for the potential pharmacological activities of ELF.

The antidiabetic function of ELF has been evaluated in animal models and humans [27], and the evidence was involved in inhibition of *α*-glucosidase and *α*-amylase in the intestine [28]. However, the antihyperglycemic effects upon ELF consumption in STZ-induced rats seem to be inconspicuous for most of the experimental period in this study. This result was different from the antidiabetic findings of ELF in a rat model of type II diabetes established by low dose STZ-injection and high-fat diet feeding [25]. It could be explained that a single injection of STZ (60 mg/kg) here nearly permanently damages pancreatic *β* cells, while the insulin secretory function is partly retained in Yang's work.

The inhibition of endogenous synthesis of NO and the elevation of vascular remodeling accelerators such as angiotensin II and endothelin-1 were considered as adverse factors in hyperglycemia. A published work demonstrated that the plasma NO contents were elevated for 1-2 weeks after STZ-treatment and afterwards reduced at four weeks [29], which was consistent with the present findings. In this study, the vasodilatation was improved upon ELF consumption, indicating the macroangiopathy in hyperglycemia could be ameliorated by ELF and as a novel therapeutic point. Moreover, the elevated NO level in serum was consistent with the ameliorative EDR in model upon ELF consumption. On the contrary, excessive accumulation of NO was considered as reactive nitrogen species to trigger oxidation of biomacromolecules in alcohol-treated rats. Administration of ELF extract (250 mg/kg) resulted in a reduction of NO levels and erythrocyte membrane lipid peroxidation [30]. Because there seems to be no acute increase of NO found at the end of the experimental process, we claim that the effects of ELF on NO levels in the present study only underline the possible function to recover endogenous NO release (still lower, compared to that in NCR) but do not focus on the radical-scavenging activity at oxidative stress.

Bioconversion by the gut microbiota and Phase I/II metabolism is pivotal to characterize the potential chemicals for cardiovascular health. In these studies, ETs were frequently considered as the potential components. However, the internal metabolite of ETs is prevailingly identified as urolithin compounds, indicating that urolithins could be beneficial* in vivo*, rather than ETs. Poor bioavailability obstructs the overestimated benefits such as free-radical-scavenging capacity* in vivo*, which is shifting attention towards to the modulatory effects of urolithins on signaling pathways involved in the vascular dysfunction. The similar standpoint is illuminated in a review illuminating the relationship between ETs and vascular health [13]. Though the vascular complications including nephropathy, oculopathy, and macrovascular complications including cardiopathy, cerebral apoplexy, and peripheral angiopathy could be alleviated upon ELF consumption, the associated molecular mechanism, especially involved in urolithin metabolites, is still rare. Therefore, we attempt to illuminate the underline mechanism involved in ellagitannin metabolite. In our study, Akt/*β*-catenin cascade in the arterial tissues of hyperglycemia rats was blocked upon ELF consumption, accompanied by the suppression of c-Myc and cyclin D1 transcription. Importantly, UroA was detectable as a metabolite in urine after ELF consumption, indicating the ETs in ELF were converted to urolithins* in vivo* and leading us to keep a watchful eye on urolithins. The findings initially put forwards a new standpoint that the benefits of ELF for diabetes complications are presumably involved in the ellagitannin metabolite urolithins. To verify this hypothesis, the potential effects of UroA on Akt/*β*-catenin signaling were assessed using two* in vitro* cell models (Figures 4 and 5). In fact, PI3K/Akt/GSK-3*β*/*β*-catenin signaling was commonly considered to promote cell proliferation and carcinogenesis [23]. Moreover, *β*-catenin and Tcf-4 play a key role in vascular remodeling by activating the c-Myc and cyclin D1 expressions, inhibiting VSMC apoptosis and promoting cell proliferation [31]. The effects of UroA on Akt/*β*-catenin signaling were assessed using an* in vitro* high glucose VSMC model. In this cell context, Akt/*β*-catenin signaling was suppressed by UroA, similar to the results in the arterial tissues above. The evidence involved in the effects of UroA on Wnt/*β*-catenin signaling was also evaluated in a LiCl-induced A7r5 cell model, which is accordant to that in our previous study on hepatoma carcinoma cells [21]. Therefore, these* in vitro* data further enhance the viewpoint that urolithins converted from ETs upon ELF consumption could be considered as beneficial substances for vascular function in hyperglycemia.

## 5. Conclusion

In the present study, the protective effects of ELF on STZ-induced VSMC dysfunction in rats were discovered for the first time. The correlation between ellagitannin metabolite UroA and HG-induced VSMC proliferation was also discussed in A7r5 cells. The primary finding of this study is that vasomotoricity damaged by STZ was improved upon ELF consumption. Moreover, the phosphorylation of Akt, accumulation of *β*-catenin, and transcription of c-Myc and cyclin D1 were suppressed, in both the artery of hyperglycemia rats and the HG-induced VSMC proliferation model upon ELF consumption or by UroA treatment, respectively. Furthermore, combining evidence from the component analysis in fruits and the metabolite identification in rats, urolithins derived from ETs in ELF were considered to be crucial for preventing VSMC dysfunction in STZ-induced hyperglycemia. In brief, we cautiously suggest that Akt/*β*-catenin signaling in hyperglycemia aortas is alleviated upon ELF consumption, which possibly attributes to, at least partly, urolithins derived from ETs in ELF.

## Abbreviations

ROS: Reactive oxygen species
NO: Nitric oxide
EDR: Endothelium-dependent relaxation
VSMCs: Vascular smooth muscle cells
ETs: Ellagitannins
ELF: Emblic leafflower fruits
UroA: Urolithin A
STZ: Streptozocin
Ach: Acetylcholine
PE: Phenylephrine
SNP: Sodium nitroprusside
NCR: Normal control rats
HGR: Hyperglycemic rats
LD: Low dose
MD: Median dose
HD: High dose
HG: High glucose
EA: Ellagic acid.

## Supplementary Materials

Supplementary 1 eFigure 1: HPLC-DAD chromatograms of the lyophilized powder of emblic leafflower fruit at 280 nm and the UV spectra (full wave scanning) of putranjivain A (A), chebulagic acid (B), and ellagic acid (C).

Supplementary 2 eTable 1: body weight (BW) (g) and blood glucose (BG) (mM) in (1) normal control rats (NCR); (2) hyperglycemic rats (HGR); (3) hyperglycemic rats treated with low dose of emblic leafflower fruit (LD); (4) hyperglycemic rats treated with median dose (MD); (5) hyperglycemic rats treated with high dose (HD).

Supplementary 3 eTable 2: primers used in real-time PCR.

## References

[1] Yechoor V., Chan L. (2005). Gene therapy progress and prospects: Gene therapy for diabetes mellitus. *Gene Therapy*.

[2] Grundy S. M., Benjamin I. J., Burke G. L. (1999). Diabetes and cardiovascular disease: a statement for healthcare professionals from the american heart association. *Circulation*.

[3] Lacigova S., Brozova J., Cechurova D., Tomesova J., Krcma M., Rusavy Z. (2016). The influence of cardiovascular autonomic neuropathy on mortality in type 1 diabetic patients; 10-year follow-up. *Biomedical Papers*.

[4] Schalkwijk C. G., Stehouwer C. D. A. (2005). Vascular complications in diabetes mellitus: the role of endothelial dysfunction. *Clinical Science*.

[5] Chen S., Evans T., Mukherjee K., Karmazyn M., Chakrabarti S. (2000). Diabetes-induced myocardial structural changes: Role of endothelin-1 and its receptors. *Journal of Molecular and Cellular Cardiology*.

[6] Ding Y., Vaziri N. D., Coulson R., Kamanna V. S., Roh D. D. (2000). Effects of simulated hyperglycemia, insulin, and glucagon on endothelial nitric oxide synthase expression. *American Journal of Physiology-Endocrinology and Metabolism*.

[7] Yam M. F., Tan C. S., Ahmad M., Shibao R. (2016). Mechanism of vasorelaxation induced by eupatorin in the rats aortic ring. *European Journal of Pharmacology*.

[8] Li H., Peng W., Zhuang J. (2013). Vaspin attenuates high glucose-induced vascular smooth muscle cells proliferation and chemokinesis by inhibiting the MAPK, PI3K/Akt, and NF-*κ*B signaling pathways. *Atherosclerosis*.

[9] Korkaya H., Paulson A., Charafe-Jauffret E. (2009). Regulation of mammary stem/progenitor cells by PTEN/Akt/*β*-catenin signaling. *PLoS Biology*.

[10] Haider U. G. B., Sorescu D., Griendling K. K., Vollmar A. M., Dirsch V. M. (2002). Resveratrol suppresses angiotensin II-induced Akt/protein kinase B and p70 S6 kinase phosphorylation and subsequent hypertrophy in rat aortic smooth muscle cells. *Molecular Pharmacology*.

[11] Liu D., Cui W., Liu B. (2014). Atorvastatin protects vascular smooth muscle cells from TGF-*β*1-stimulated calcification by inducing autophagy via suppression of the *β*-catenin pathway. *Cellular Physiology and Biochemistry*.

[12] Ghosh D., Scheepens A. (2009). Vascular action of polyphenols. *Molecular Nutrition & Food Research*.

[13] Larrosa M., García-Conesa M. T., Espín J. C., Tomás-Barberán F. A. (2010). Ellagitannins, ellagic acid and vascular health. *Molecular Aspects of Medicine*.

[14] Yang B., Kortesniemi M., Liu P., Karonen M., Salminen J.-P. (2012). Analysis of hydrolyzable tannins and other phenolic compounds in emblic leafflower (*Phyllanthus emblica* L.) fruits by high performance liquid chromatography-electrospray ionization mass spectrometry. *Journal of Agricultural and Food Chemistry*.

[15] Dobroslawa B., Kasimsetty S. G., Khan S. I., Daneel F. (2009). Urolithins, intestinal microbial metabolites of pomegranate ~ellagitannins, exhibit potent antioxidant activity in a cell-based assay. *Journal of Agricultural and Food Chemistry*.

[16] Fuhrman B., Lavy A., Aviram M. (1995). Consumption of red wine with meals reduces the susceptibility of human plasma and low-density lipoprotein to lipid peroxidation. *American Journal of Clinical Nutrition*.

[17] Bolzán A. D., Bianchi M. S. (2002). Genotoxicity of Streptozotocin. *Mutation Research*.

[18] Cai S., Khoo J., Mussa S., Alp N. J., Channon K. M. (2005). Endothelial nitric oxide synthase dysfunction in diabetic mice: Importance of tetrahydrobiopterin in eNOS dimerisation. *Diabetologia*.

[19] Zhang F., Ram J. L., Standley P. R., Sowers J. R. (1994). 17beta-estradiol attenuates voltage-dependent Ca2+ currents in A7r5 vascular smooth muscle cell line. *American Journal of Physiology*.

[20] Zhou B., Wang J., Zheng G., Qiu Z. (2016). Methylated urolithin A, the modified ellagitannin-derived metabolite, suppresses cell viability of DU145 human prostate cancer cells via targeting miR-21. *Food and Chemical Toxicology*.

[21] Wang Y., Qiu Z., Zhou B. (2015). In vitro antiproliferative and antioxidant effects of urolithin A, the colonic metabolite of ellagic acid, on hepatocellular carcinomas HepG2 cells. *Toxicology in Vitro*.

[22] González-Sarrías A., Giménez-Bastida J. A., García-Conesa M. T. (2010). Occurrence of urolithins, gut microbiota ellagic acid metabolites and proliferation markers expression response in the human prostate gland upon consumption of walnuts and pomegranate juice. *Molecular Nutrition & Food Research*.

[23] Son Y.-O., Wang L., Poyil P. (2012). Cadmium induces carcinogenesis in BEAS-2B cells through ROS-dependent activation of PI3K/AKT/GSK-3*β*/*β*-catenin signaling. *Toxicology and Applied Pharmacology*.

[24] Poltanov E. A., Shikov A. N., Dorman H. J. D. (2009). Chemical and antioxidant evaluation of Indian gooseberry (*Emblica officinalis* Gaertn., syn. *Phyllanthus emblica* L.) supplements. *Phytotherapy Research*.

[25] Yang B., Liu P. (2014). Composition and biological activities of hydrolyzable tannins of fruits of phyllanthus emblica. *Journal of Agricultural and Food Chemistry*.

[26] Majeed M., Bhat B., Jadhav A. N., Srivastava J. S., Nagabhushanam K. (2009). Ascorbic acid and tannins from *Emblica officinalis* Gaertn. Fruits—a revisit. *Journal of Agricultural and Food Chemistry*.

[27] D'Souza J. J., D'Souza P. P., Fazal F., Kumar A., Bhat H. P., Baliga M. S. (2014). Anti-diabetic effects of the Indian indigenous fruit Emblica officinalis Gaertn: Active constituents and modes of action. *Food & Function*.

[28] Nampoothiri S. V., Prathapan A., Cherian O. L., Raghu K. G., Venugopalan V. V., Sundaresan A. (2011). In vitro antioxidant and inhibitory potential of Terminalia bellerica and Emblica officinalis fruits against LDL oxidation and key enzymes linked to type 2 diabetes. *Food and Chemical Toxicology*.

[29] Oyadomari S., Gotoh T., Aoyagi K., Araki E., Shichiri M., Mori M. (2001). Coinduction of endothelial nitric oxide synthase and arginine recycling enzymes in aorta of diabetic rats. *Nitric Oxide: Biology and Chemistry*.

[30] Kumaran A., Karunakaran R. J. (2006). Nitric oxide radical scavenging active components from *Phyllanthus emblica* L.. *Plant Foods for Human Nutrition*.

[31] Wang X., Xiao Y., Mou Y., Zhao Y., Blankesteijn W. M., Hall J. L. (2002). A role for the *β*-catenin/T-cell factor signaling cascade in vascular remodeling. *Circulation Research*.

