# Supplementary figures and images for: Emblic Leafflower (Phyllanthus emblica L.) Fruits Ameliorate Vascular Smooth Muscle Cell Dysfunction in Hyperglycemia: An Underlying Mechanism Involved in Ellagitannin Metabolite Urolithin A
Source: Evid Based Complement Alternat Med. 2018 Mar 6;2018:8478943. doi: 10.1155/2018/8478943 (PMC5859803; doi:10.1155/2018/8478943)

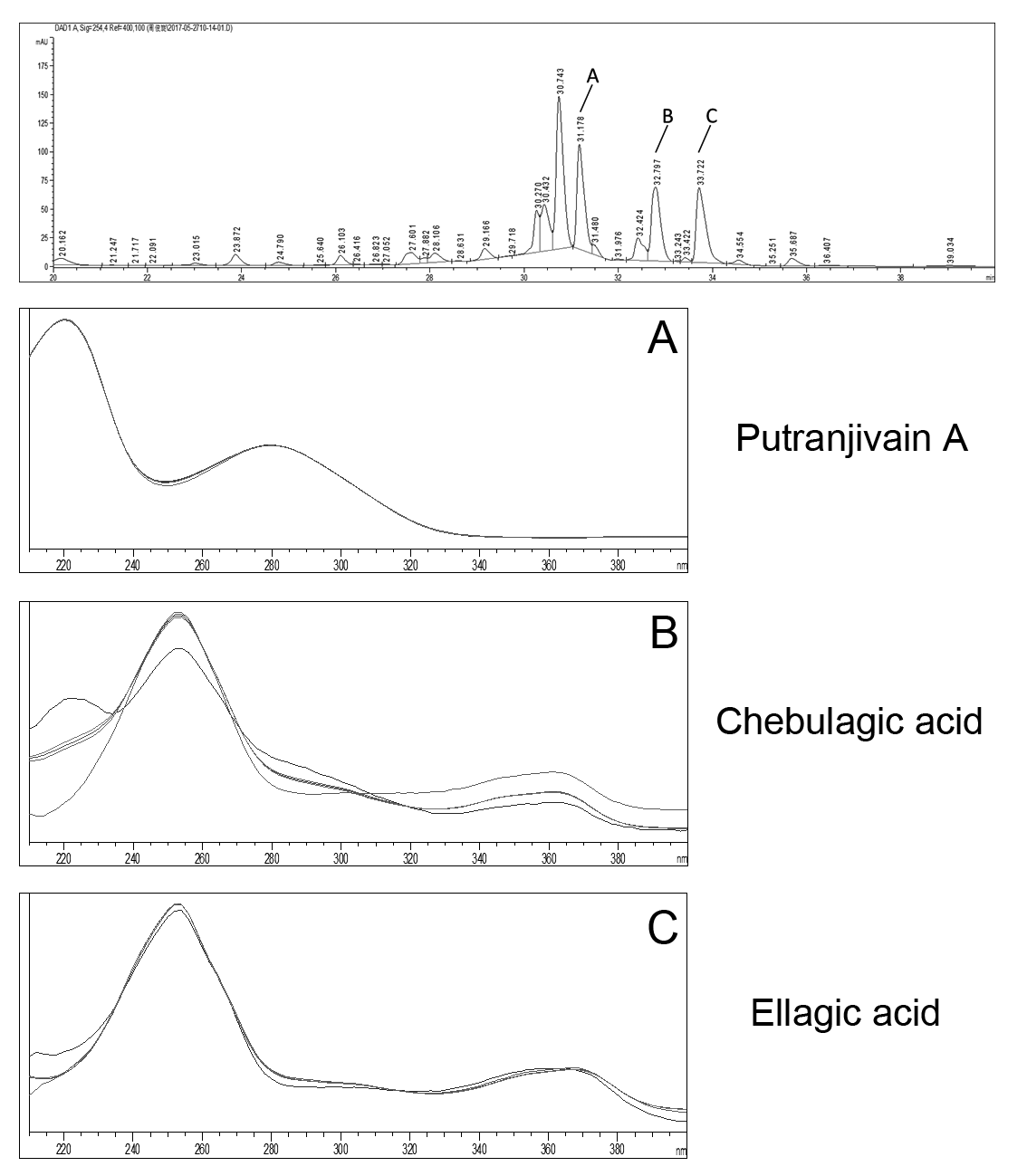

Supplement: Supplementary 1 — eFigure 1: HPLC-DAD chromatograms of the lyophilized powder of emblic leafflower fruit at 280 nm and the UV spectra (full wave scanning) of putranjivain A (A), chebulagic acid (B), and ellagic acid (C). [file 8478943.f1.tif]
