# Supplementary material for: Emblic Leafflower (Phyllanthus emblica L.) Fruits Ameliorate Vascular Smooth Muscle Cell Dysfunction in Hyperglycemia: An Underlying Mechanism Involved in Ellagitannin Metabolite Urolithin A
Source: Evid Based Complement Alternat Med. 2018 Mar 6;2018:8478943. doi: 10.1155/2018/8478943 (PMC5859803; doi:10.1155/2018/8478943)
Supplement: Supplementary 2 — eTable 1: body weight (BW) (g) and blood glucose (BG) (mM) in (1) normal control rats (NCR); (2) hyperglycemic rats (HGR); (3) hyperglycemic rats treated with low dose of emblic leafflower fruit (LD); (4) hyperglycemic rats treated with median dose (MD); (5) hyperglycemic rats treated with high dose (HD). [file 8478943.f2.docx]

**eTable 1** Body weight (BW) (g) and blood glucose (BG) (mM) in (1) normal control rats (NCR); (2) hyperglycemic rats (HGR); (3) hyperglycemic rats treated with low dose of emblic leafflower fruit (LD); (4) hyperglycemic rats treated with median dose (MD); (5) hyperglycemic rats treated with high dose (HD).

|  | **NCR** | **HGR** | **HGR+LD** | **HGR+MD** | **HGR+HD** |
| --- | --- | --- | --- | --- | --- |
| **BW (5W)** | 344.67±15.98 | 282.33±37.08# | 299.67±29.65# | 325.00±21.60 | 341.17±19.96 |
| **BG (3W)** | 7.43±0.34 | 30.36±1.17# | 28.78±2.27# | 27.95±2.68# | 21.45±0.92#* |
| **BG (5W)** | 8.17±0.41 | 29.25±0.72# | 28.36±4.17# | 29.52±2.02# | 27.36±3.17# |

Mean±S.D. # P<0.05 *vs.* NCR; * P<0.05 *vs.* HGR.
