# Supplementary material for: Emblic Leafflower (Phyllanthus emblica L.) Fruits Ameliorate Vascular Smooth Muscle Cell Dysfunction in Hyperglycemia: An Underlying Mechanism Involved in Ellagitannin Metabolite Urolithin A
Source: Evid Based Complement Alternat Med. 2018 Mar 6;2018:8478943. doi: 10.1155/2018/8478943 (PMC5859803; doi:10.1155/2018/8478943)
Supplement: Supplementary 3 — eTable 2: primers used in real-time PCR. [file 8478943.f3.docx]

**eTable 2** Primers used in realtime PCR.

| **Gene** | **Forward primer 5′ to 3′** | **Reverse primer 5′ to 3′** |
| --- | --- | --- |
| **c-Myc** | TGAACACAGCGAATGTTTCC | TTAGGAGCGCTCAGGTCTGT |
| **Cyclin D1** | CAGGTTGGA CAGTTCACAGG | ACAGCTGGAGTTGGATGGAC |
| **β-actin** | CCTGGCACCCAGCACAAT | GGGCCGGACTCGTCATACT |
